# Evaluating convolutional neural networks using residual blocks and global average pooling techniques for predicting sediment concentration

**DOI:** 10.1038/s41598-025-19161-w

**Published:** 2025-10-09

**Authors:** Cheng-Chia Huang, Che-Cheng Chang, Chiao-Ming Chang

**Affiliations:** 1 Department of Water Resources Engineering and Conservation, Feng Chia University, Taichung City, Taiwan; 2 Department of Information Engineering and Computer Science, Feng Chia University, Taichung City, Taiwan

**Keywords:** Sediment concentration (SC), Convolutional neural network (CNN), Residual block (RB), Global average pooling (GAP), Real-time, Environmental sciences, Civil engineering

## Abstract

Monitoring sediment concentration (SC) is a challenge in water resource management due to environmental complexities and sensor limitations. Hence, developing a monitoring technology that is easy to operate, high precision, and cost-effective is forward-looking. This research is based on convolutional neural networks and further introduces the concepts of residual block and global average pooling layer to develop a prediction model (CNN-SCP, Convolutional Neural Networks-Sediment Concentration Prediction) to predict sediment concentration. Here, residual blocks (RB) mitigate the vanishing gradient problem and enhance feature propagation for improved stability and convergence. Also, the global average pooling layer reduces parameters, prevents overfitting, and enhances generalization by replacing fully connected layers with spatial averaging, improving model robustness and efficiency. As a result, the proposed model improves the performance of primitive convolutional neural networks, which are apparently better than the existing ones in the literature regarding many metrics, such as MAE is 115.42 and 263.67, MAPE is 6.38 and 14.67, RMSE is 134.24 and 294.51, and CC is 0.97 and 0.90, respectively, by comparing the 5RBs and previous. These results demonstrate the potential of CNN-SCP for future real-time sediment monitoring and early warning system deployment.

## Introduction

Suspended solid refers to material particles that are present in a liquid medium but are not dissolved, and the source of the particle includes a wide range of materials such as sediment, silt, organic matter, and other particulate matter^1–3^. In the water resources management field, the suspended sediment of watersheds and rivers has been investigated extensively to understand the worldwide situation in the past decades^4–6^. Until today, understanding the physical phenomena of suspended sediment transport, attempting to quantify the sediment concentration (SC), and implementing relevant response strategies to mitigate SC-related issues, such as, debris flow, reservoir deposition, and river sedimentation, remain the goals of water resources and disaster management around the world^7–10^.

Several pieces of research focus on the calculation of the SC by using empirical formulas. However, These models rely heavily on parameter calibration^11–15^. Numerical simulations provide detailed spatial-temporal modeling but also require extensive field data and assumptions^16–20^. It is worth noting that empirical formulas and numerical simulations require many assumptions and field data to increase their credibility. Unfortunately, field data is often limited by environmental factors such as heavy flood or huge sedimentation and is difficult to obtain in sufficient quantities. As a result, the availability of consistent and accurate field data remains a persistent challenge in sediment monitoring. The development of stable monitoring technologies not only enhances data accuracy but also provides more reliable datasets for empirical formulas and numerical models. Acoustic Doppler Current Profilers (ADCP) is an updated technology that provides SC information using the acoustic backscatter strength approach. It is considered to provide the spatial distribution of SC more conveniently in the field site^21–25^. Another method for measuring SC is Time Domain Reflectometry (TDR), which estimates concentration based on the relationship between various dielectrics and sediment particles. TDR has been utilized for sediment observations in field sites and is suitable in high-concentration areas^26–30^. RSS (Remote Sensing Satellites) is a method that overcomes spatial limitations and observes large areas. By analyzing the characteristics of different layers, the water flow patterns and concentration variation could be grasped to estimate the SC^31–35^. Unmanned Aerial Vehicles (UAVs) are an emerging technology that enables rapid obtaining different images. They can overcome the time-consuming and labor-intensive drawbacks of traditional methods, and now, UAVs are widely used to analyze the SC distributions in surface water bodies^36–40^. Analyzing the above methods, ADCP faces difficulties in deployment on high-velocity stream surfaces, such as heavy rainfall and typhoon events; TDR exhibits lower accuracy in low-concentration environments compared to high-concentration periods; RSS may encounter cloud cover requiring image restoration, leading to issues in obtaining imagery at crucial time points; UAVs must overcome flight challenges during strong winds and heavy rain. In conclusion, developing a monitoring technology that is easy to operate, high precision, and cost-effective is forward-looking.

Recently, the authors in^41^ introduce a new approach, which employs a classification based on Convolutional Neural Network (CNN) and then incorporates the ensemble architecture to evaluate its performance. Particularly, the process of partitioning the raw image into multiple image segments is realized first. Then, they average the former to get the result for each raw image. Such can tolerate the problem of uneven suspended solids. This study brings an interesting concept on how to deal with SC measurement. However, since their CNN architecture is a general design without effective and functional components, it can only cope with larger measurement intervals that may not be enough for applications in practice. Consequently, in this paper, we incorporate several critical components to improve the performance of CNN so that ours can deal properly with smaller measurement intervals and possess better accuracy. For example, the concept of Residual Block (RB)^42^ is utilized to alleviate the vanishing gradient problem, improve feature propagation, and reduce overfitting. Similarly, instead of the usage of flatten layers, we introduce the notion of Global Average Pooling (GAP)^43^, which can facilitate parameter reduction as well as class activation mapping. Noteworthily, the latter advantage of GAP exactly fits our design for its integration of classification into regression. Notably, some studies in the literature rely on specialized equipment to implement their neural network estimations, which can limit the scalability and broader applicability of the research. For example, the authors in^44^ estimate the number of soil components by passing laser light through the beaker and evaluating the changes in the magnitude of light. Moreover, the authors in^45^ utilize broadband sensors that offer lower spectral resolution but higher spatial and temporal resolution. However, such cameras are not feasible for widespread deployment across all monitoring areas, especially for IoT applications. In contrast, our method leverages simple RGB images captured under standard lighting conditions, significantly reducing hardware requirements and improving applicability in real-world scenarios.

Although previous studies, such as^41^, have demonstrated the potential of CNNs for sediment concentration estimation, existing models often target larger measurement intervals (500 ppm), and their general architectures have limited capability in processing subtle variations in image features. This restricts their applicability in real-time early warning systems that demand higher precision. Furthermore, many methods^44,45^ rely on specialized optical equipment, limiting their scalability. Consequently, a clear gap exists in the literature for a deep learning model that can achieve finer prediction intervals (250 ppm) using only standard RGB images, while being both cost-effective and efficient. To address this gap, this study proposes and evaluates an improved CNN architecture, named CNN-SCP (Convolutional Neural Networks-Sediment Concentration Prediction). The primary contributions of this work are listed below.

1. A Novel Model Architecture: This study proposes a new CNN architecture, named CNN-SCP, which is the first to integrate Residual Blocks (RB) and a Global Average Pooling (GAP) layer for sediment concentration prediction from standard RGB images. This design enhances feature propagation and reduces the risk of overfitting.
2. Superior Prediction Accuracy at Finer Granularity: The proposed model is empirically demonstrated to achieve significantly higher accuracy (98.37% on the testing set) at a finer prediction resolution (250 ppm intervals) compared to the baseline model, making it more suitable for practical applications.
3. Demonstrated Practicality and Low-Cost Potential: This study validates a technical approach for high-precision monitoring that relies only on low-cost cameras and standard lighting, providing an empirical basis for the future deployment of economical and efficient real-time sediment monitoring and early warning systems.

### Neural network framework

A Neural Network (NN) is a model derived from Machine Learning (ML) and is inspired by the structure of biological neural networks. Thanks to its inherent characteristics, NN can be integrated with various technologies to address extensive issues such as image classification, speech recognition, optimization, and so forth^46–48^. Its versatility, efficacy, and scalability make it an invaluable tool in computational problem-solving.

Next, a CNN is a type of neural network. Its use of convolutional operations significantly reduces the number of trainable parameters and computational complexity of traditional neural networks, especially for image processing. This structure draws inspiration from the organization of the animal visual cortex’s connectivity patterns^47,48^.

### Design concept

The authors in^41^ use a general design of CNN to extract features from an image. However, since their CNN architecture is a general design without effective and functional components, it can only cope with larger measurement intervals that may not be enough for applications in practice. This motivates us to modify the CNN architecture in order to improve the extraction performance for SC prediction.

Consequently, several critical components are introduced into our CNN model. Firstly, the concept of RB is utilized to alleviate the vanishing gradient problem, improve feature propagation, and reduce overfitting. The key concept is the shortcut connection or skip connection that allows the input to bypass one or more layers and be added directly to the output of these layers. More specifically, an RB consists of several various layers. After these operations, instead of moving to the next set of layers, the input is added back to the output of these layers. This helps to mitigate the vanishing gradient problem, as it provides an alternative path during backpropagation. Furthermore, it also learns more adjustments needed on the input itself by improving information flow.

Similarly, instead of the usage of flatten layers, we introduce the notion of GAP to our design, which can facilitate parameter reduction as well as class activation mapping. Instead of flattening the feature maps and connecting them to fully connected layers, GAP calculates the average of every feature map. More specifically, if we have a feature map of size w x h (width x height), GAP computes the average of all w x h values, resulting in a single value. This operation is performed for every feature map independently, transforming the set of feature maps into a flat vector. Hence, it can reduce the total number of trainable parameters in the model and decrease the risk of overfitting. Noteworthily, its advantage exactly fits our design for its integration of classification into regression. Accordingly, our new model can deal properly with smaller measurement intervals and possess better accuracy.

### Proposed technology

**Fig. 1:**
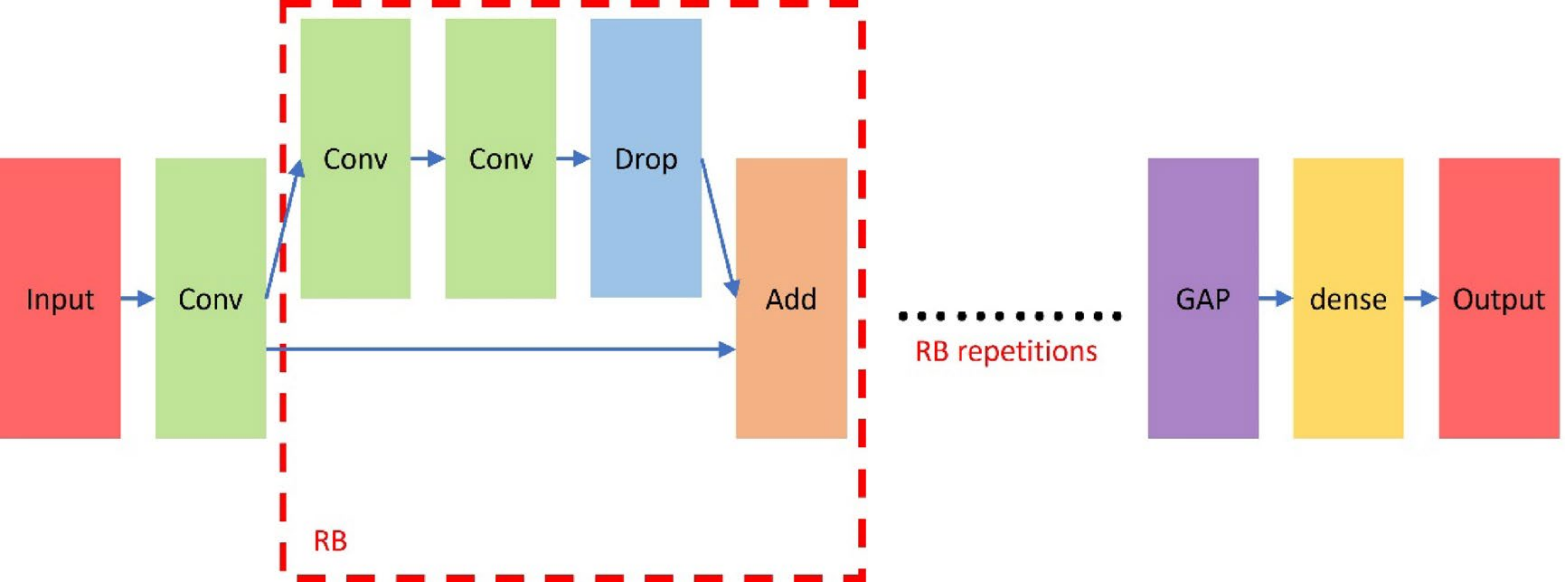
The design of Proposed NN.

Based on the above discussions, we design a new CNN architecture for our experiments. In Fig. 1, the image is inputted and passed through a convolutional layer first. Then, an RB is adopted, where there are three convolutional layers, one dropout layer, and one add layer successively. Noteworthily, we give the RB repetitions to repeat the RB several times to evaluate the performance further, i.e., three, four, and five times. Finally, a GAP layer and a dense layer are used to facilitate the sample classification before the output layer. Notice that the model receives input as a 224 × 224 RGB image, represented by neurons corresponding to the image’s pixel values across three color channels. The final output layer consists of 32 neurons, each representing a distinct concentration category ranging from 0 to 8000 ppm in 250 ppm intervals.

### Parameter setting

The specification of the experimental computer shows below:

(i) CPU: Intel Xeon E5-2667V4;
(ii) RAM: 64G;
(iii) GPU: GeForce GTX 1080 Ti 11G;
(iv) Python: 3.8.18;
(v) TensorFlow: 2.10.0.

The model parameters and the experimental independent variables are listed in Table 1. A batch size of 4 is chosen to introduce gradient noise, which can help improve generalization and facilitate more frequent parameter updates. A dropout rate of 0.2 is applied to prevent overfitting while preserving model capacity. The learning rate is set to 0.001, a widely accepted starting point that balances convergence speed and stability when using adaptive optimizers such as Adam. The Adam optimizer was selected for its proven effectiveness in CNN training, with standard parameters β₁ = 0.9, β₂ = 0.999, and ε = 10^− 7^ to ensure stable optimization. Categorical cross-entropy is the loss function, which is appropriate for multi-class image classification tasks. The model is trained for up to 100 epochs, and the epoch yielding the best validation performance is selected to avoid overfitting and ensure optimal generalization. Next, the number of RB repetitions is utilized to repeat the RB several times to evaluate the performance further, i.e., three, four, and five times.

**Table 1:** Adopted parameters.

Name	Description
Batch size	4
Dropout	0.2
Learning rate	0.001
Optimizer	Adam
β₁	0.9
β₂	0.999
ε	10^− 7^
Loss function	Categorical Cross-Entropy
Epochs	100 epochs and the best epoch
Number of RB repetitions	3, 4, and 5

## Dataset and metric

### Image material

**Fig. 2:**
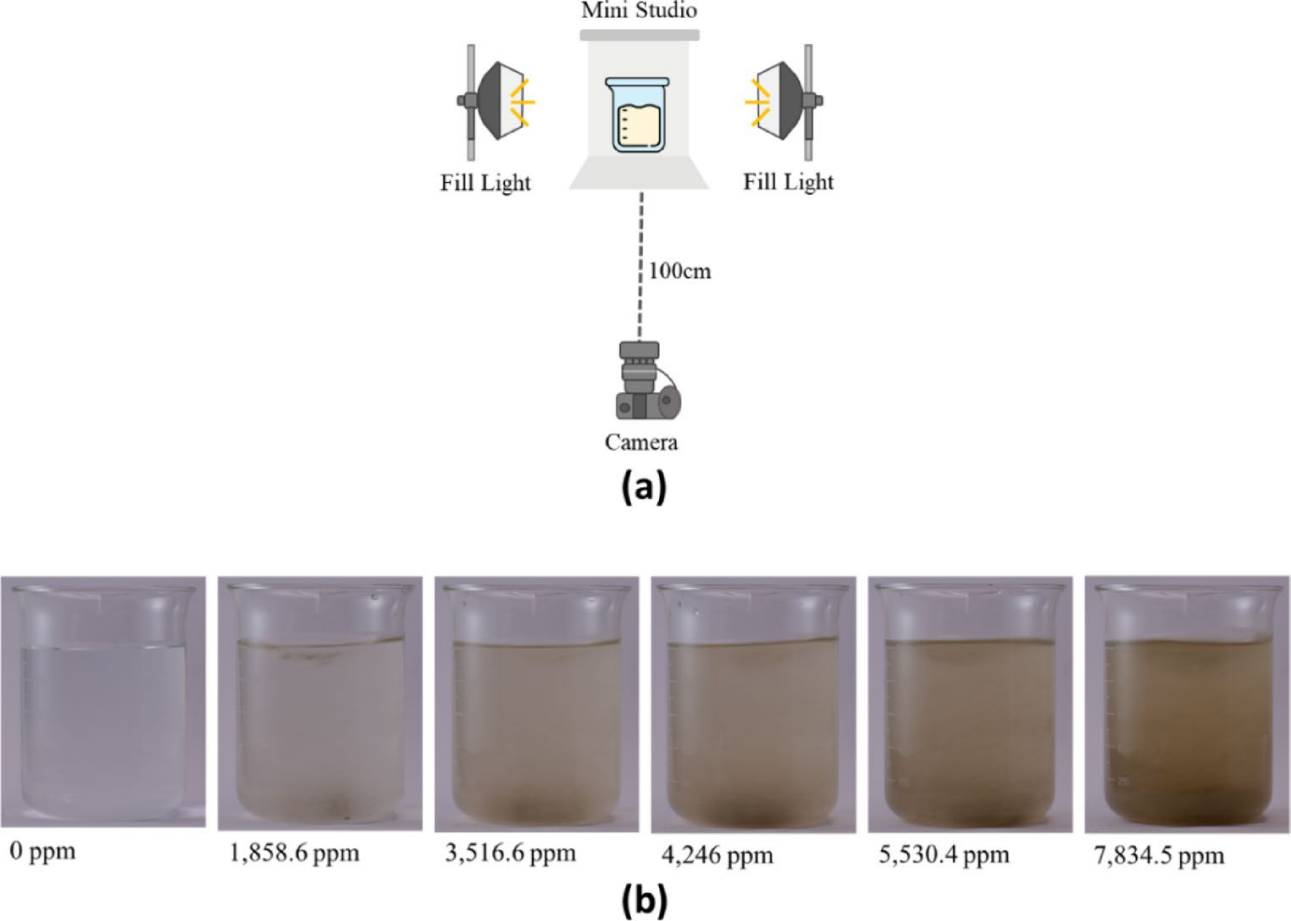
The experimental setup and some samples.

In this study, all experimental photographs are captured using FUJIFILM X-S10, an APS-C (Advanced Photo System Classic) camera. The camera setting parameters include (1) Aperture: f/8; (2) Shutter speed: 1/25 second; (3) ISO: 200; (4) Focal length: 35 mm; (5) Color temperature: 4000; (6) Manual mode; (7) Picture resolution: 6240*4160. Next, the experimental setup is depicted in Fig. 2 (a). The camera is positioned horizontally relative to the sample in the film studio, maintaining a distance of 100 cm between them. Subsequently, two fill lights are horizontally arranged on both sides of the film studio to mitigate contrast, aligning with the dynamic range of the photograph. This adjustment aims to capture an equivalent level of detail as perceived by humans.

Except for those expensive survey instruments, most devices have an interval of 500 ppm, and this is also adopted in^41^. Hence, we set a further goal to design a classifier for the interval of 250 ppm. In particular, this study prepared 60 samples with a well-mixed pre-process for both training and testing procedures. The concentration of 60 samples is from 100 to 7,800 ppm, in random increments of 100–150 ppm. Some samples with various mixtures of sediment and water body are given in Fig. 2 (b), and those are 0 ppm, 1,858.6 ppm, 3,516.6 ppm, 4,246 ppm, 5,530.4 ppm, and 7,834.5 ppm from left to right. Accordingly, the output layer includes 32 neurons to classify samples into 32 categories with 250 ppm intervals from 0 to 8,000 ppm. Next, to avoid creating an overly complex network and the mixed issue of the settling of suspended sands in water, we choose not to utilize entire images as training or testing samples. Instead, we adopt the sliding window technique with a stride = 10 to segment the images into smaller segments (224*224 pixels), generating more manageable samples while maintaining network size. This setting follows the configuration used in the method proposed in^41^, which serves as the baseline for our study. This also ensures that the number of samples is sufficient for our experiments. Using the sliding window technique, we generate 277,440 sub-images. Note that these sub-images only cover the water regions, rather than the entire original image. These sub-images are then divided into 80% training data and 20% testing data for the purpose of preventing overfitting.

### Evaluation metric

Since the CNN concept is utilized to predict the classifications of sub-images in this paper, numerous evaluative skills of CNN can be adopted to give comprehensive considerations as well. Via various expressions, we provide the classifying accuracy for all sub-images in the training and testing datasets. Then, we average the results of sub-images in a single raw image to predict each raw image. Next, according to the prediction of each raw image, four classical metrics will be adopted to further evaluate the performance of the different models.

Firstly, MAE stands for Mean Absolute Error. It is commonly used to evaluate the performance of a predictive model, particularly in regression analysis. MAE is calculated by taking the average of the absolute differences between the predicted values and the actual values. It provides a measure of how close the predictions are to the actual values, with lower values indicating better performance.

MAPE (Mean Absolute Percentage Error) is calculated as the absolute percentage error between actual and predicted values, averaged across all data points. A lower MAPE value indicates a more accurate prediction. As MAPE is expressed as a percentage, it remains unaffected by the scale of data points, facilitating comparative analysis (i.e., the scale for judging accuracy remains consistent).

The third metric, RMSE (Root Mean Squared Error) is derived from the square root of the mean squared error, making it easily interpretable. RMSE computes the square of the differences between actual and predicted values, averages them, and then takes the square root. This process penalizes large errors more severely due to the squaring operation, rendering RMSE sensitive to outliers. Consequently, a lower RMSE signifies a more accurate model.

The correlation coefficient (CC) is a statistical measure that quantifies the strength and direction of the relationship between the predicted and actual values. It ranges from − 1 to 1. When the CC equals 1, it indicates a perfect positive linear relationship, meaning that as one variable increases, the other variable also increases proportionally. On the contrary, a perfect negative linear relationship, meaning the CC equals − 1. Besides, the CC equals 0, it indicates no linear relationship between the two values.

These metrics are commonly used in assessing the performance of predictive models, with each providing unique insights into the accuracy and predictive power of the proposed model. The above formula of MAE, MAPE, RMSE, and CC show in Eq. (1) to (4).

1 $$MAE = \frac{1}{n}\sum\nolimits_{{i = 1}}^{n} {\left| {(P_{i} - A_{i} )} \right|}$$

2 $$MAPE = \frac{1}{n}\sum\nolimits_{{i = 1}}^{n} {\left| {\frac{{(P_{i} - A_{i} )}}{{A_{i} }}} \right|} \times 100\%$$

3 $$RMSE = \sqrt {\frac{1}{n}\sum\nolimits_{{i = 1}}^{n} {(P_{i} - A_{i} )^{2} } }$$

4 $$CC = \frac{{\sum\nolimits_{{i = 1}}^{n} {(P_{i} - \bar{P})(A_{i} - \bar{A})} }}{{\sqrt {\sum\nolimits_{{i = 1}}^{n} {(P_{i} - \bar{P})^{2} \sum\nolimits_{{i = 1}}^{n} {(A_{i} - \bar{A})^{2} } } } }}$$

where *n* is the sample number; *P* and *A* are the prediction and actual value, respectively; $\bar {P}$ and $\bar {A}$ are the means of prediction and actual value, respectively.

## Model performance

This study decided to use the model from^41^ as the primary baseline is grounded in its published, comprehensive benchmarking. That study demonstrated that their proposed model outperformed several classical and deep learning models, including SVM, VGG19, and ResNet50, on the task of sediment concentration prediction. Therefore, the work in^41^ represents the most competitive and relevant state-of-the-art for this specific application. By comparing directly against it, we can most effectively validate the incremental improvements brought by our novel architecture.

### Training and testing phase

**Fig. 3:**
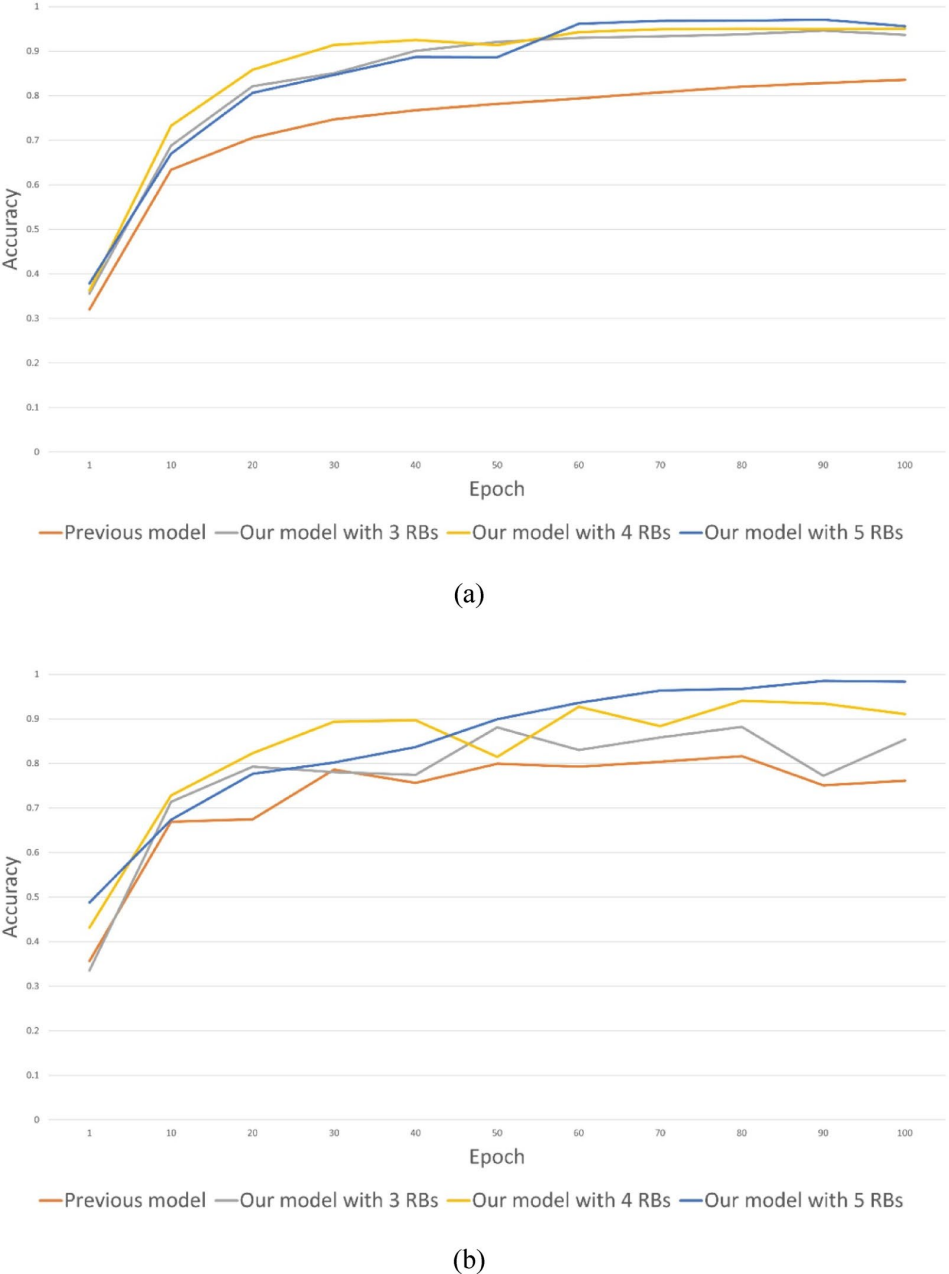
Accuracy of training procedure using (**a**) training data; (**b**) testing data.

Here, we start to evaluate our CNN design. The results are shown in Fig. 3, where the previous model mentioned in both figures is proposed in^41^. Note that we adopt the method proposed in^41^ as our primary baseline for performance comparison. This is because^41^ has already conducted comprehensive evaluations against several well-known classifiers, including Support Vector Machines (SVM), VGG19, and ResNet50, and demonstrate superior performance over these models. Given this prior benchmarking, our focus is directed toward comparing our proposed model with the method in^41^.

According to these results, it is obvious that our models are better than the model proposed in^41^, no matter how many RBs are adopted in our models. Particularly, while utilizing the training data to realize the validation test, the accuracy of all our models is higher than 90% at the end of the training procedure. While utilizing the testing data to realize the validation test, the accuracy of our models with 4 and 5 RBs is also higher than 90% at the end of the training procedure.

Next, consider the best epoch, which is an experiment with various epochs to find the optimal number that achieves the best performance via the testing dataset, our model with 5 RBs possesses the best accuracy among all other methods, i.e., 98.37%. Noteworthily, since the testing dataset is not adopted to realize the training procedure, the accuracy of the best epoch shows its importance and fairness. Notice that during the training process, we save the model obtained at each epoch and evaluate its performance on the test data. This approach allows us to identify the best-performing model, which achieves optimal results and helps prevent overfitting.

### Optimization phase

**Fig. 4:**
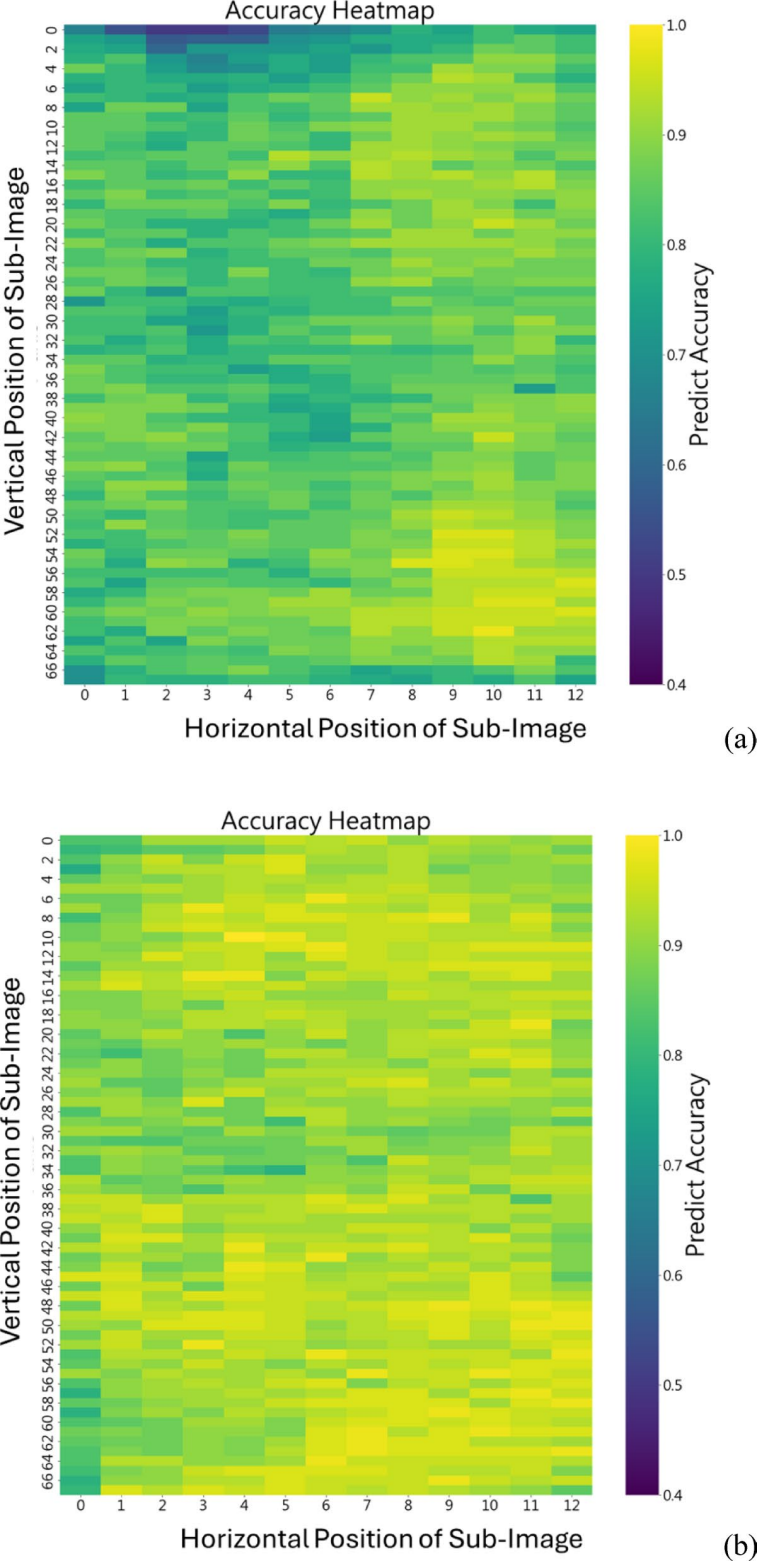

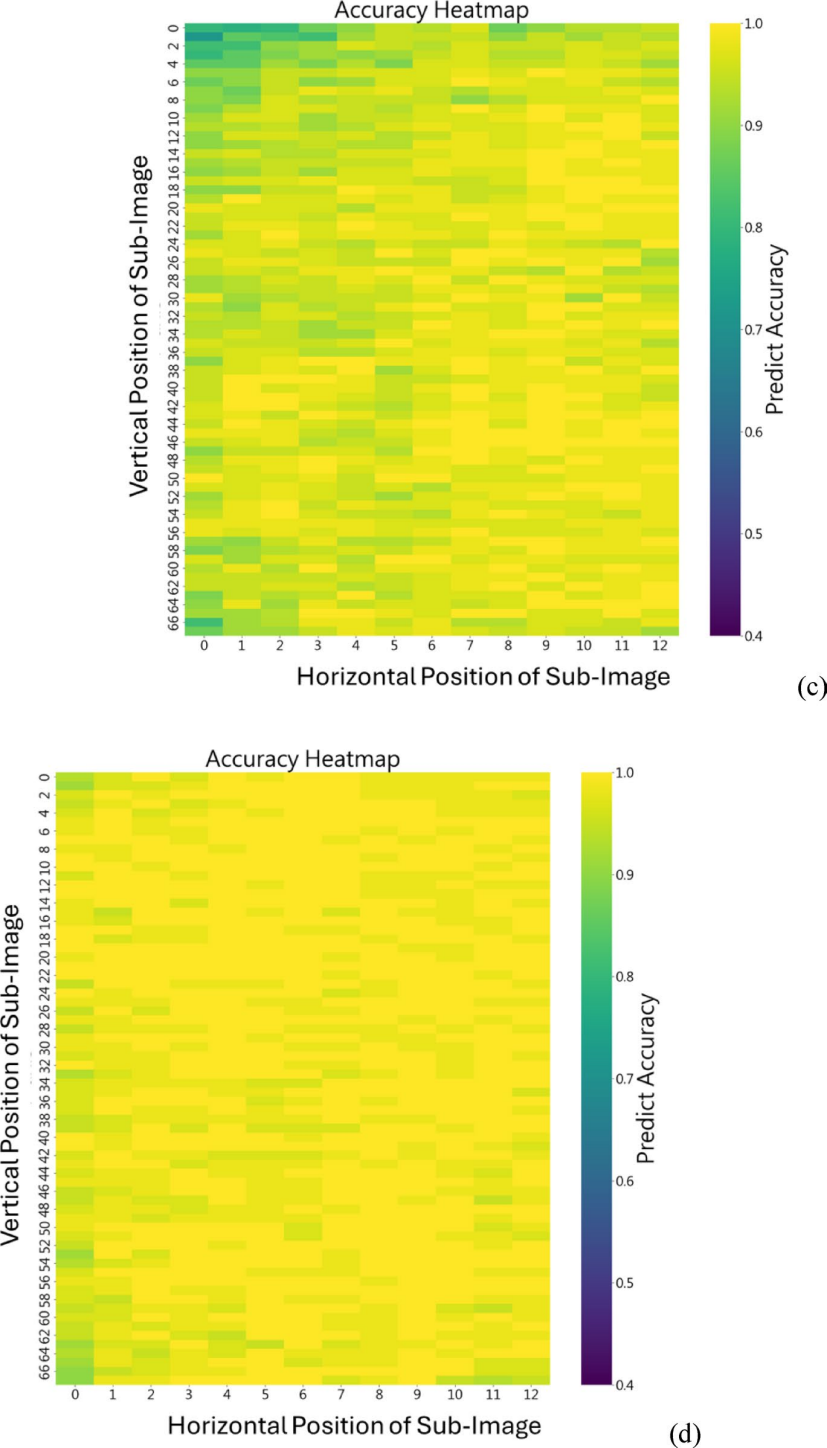
The heat map generated from (**a**) previous model; (**b**) our model with 3 RBs; (**c**) our model with 4 RBs; (**d**) our model with 5 RBs.

Next, we use the best epochs of all models to draw the heat maps to illustrate the accuracy of each sub-image from the raw image. The figures are shown in Fig. 4. Obviously, our models are better no matter where the position of the sub-images is, especially the one with 5 RBs. Furthermore, we can observe that in Fig. 4 (a), there are some sub-figures with especially lower accuracy due to the uneven mixing that causes sub-images with less distinct features. This problem is dealt with properly since our model with RB allows the input to bypass one or more layers to improve feature propagation. The model with 5 RBs is presented in Fig. 4 (d), and its testing accuracy of all sub-images is almost 100%.

On the other hand, Table 2 shows the training and predicting time. Since our models are more complex than the previous model proposed in^41^, we need more time to finish the training procedure. Note that the time-consuming problem does not exist. The prediction time is similar for each sub-image, and the difference between them is only about 0.002 s, as shown in Table 2.

**Table 2:** The training and predicting time.

	Previous model	3 RBs	4 RBs	5 RBs
Training time	≈ 9 h	≈ 7 days and 21 h	≈ 10 days and 6 h	≈ 9 days and 18 h
Predicting time	0.0471 s	0.0478 s	0.0477 s	0.0490 s

Moreover, to evaluate the contribution of residual blocks, we experiment with models incorporating 3, 4, and 5 RBs. The results show that increasing the number of RBs leads to notable improvements in prediction accuracy. Although the performance gains between 4 and 5 RBs are smaller, the model with 5 RBs still achieves the best overall accuracy. These findings suggest that residual connections enhance feature propagation and improve the model’s ability to capture complex patterns in image-based sediment concentration prediction. At the same time, GAP’s effectiveness can be inferred from the overall model performance and design benefits. GAP replaces traditional fully connected layers, significantly reducing the number of trainable parameters and helping to prevent overfitting. This design choice also enables more efficient training and inference, as evidenced by the minimal increase in prediction time across deeper networks.

## Discussion

### Evaluation of CNN-SCP method

The obvious optimized performance based on the enhancement prediction model is presented in Chap. 4 by comparing the previous model. In Chap. 5, we further adopt four evaluation metrics, MAE, MAPE, RMSE, and CC, to evaluate the current model performance completely. Note that 1,000 ppm is the interval, and 8 compared groups are decided from 0 to 8000 ppm. Table 3 shows each value and we discuss below.

Regarding Mean Absolute Error (MAE), the previous model demonstrated stable and reasonable values within the concentration range of 0-7000, with the majority falling between 200 and 300 ppm. Our proposed model further optimized predictive performance. For 3 RBs, the range spans from 63.53 to 146.17, with most falling within the range of 100–150. Further analysis reveals that MAE values for 4 RBs and 5 RBs exhibit continued optimization compared to 3 RBs, albeit with a smaller magnitude. It is evident that the optimized model exhibits a significant improvement in MAE values.

MAPE can clearly indicate the absolute percentage error between actual and predicted values. All models exhibit higher values in the range of 0-1000 ppm, but the optimized model clearly outperforms the previous model. For 3RBs, the data shows a 30.05% error, which is better than the previous model’s 61.31%. The error percentages across different concentration ranges gradually decrease for all models, with the optimized model consistently staying below 10%, still outperforming the performance of the previous model.

RMSE is an alternative perspective for evaluation. The performance of the previous model ranged between 200 and 400. Regardless of whether it is 3 RBs, 4 RBs, or 5 RBs, the maximum values are all smaller than 200. Additionally, the optimized model presents the best performance in the range of 2000–3000 ppm. For 3 RBs, the minimum value reached 72, while 5 RBs could be further optimized to 63.99.

The CC is a suitable index to evaluate the relative of predicted and actual values. The previous model showed the high values, 0.86 to 0.98, in different concentration ranges. The better performance showed in range 2000–3000 ppm, the value is 0.98. The current model presents the better performance by comparing with the previous model. In 3RBs, the range of CC is between 0.94 and 0.97. In 4RBs and 5RBs, the CC further improves to 0.97, and the highest value reaches 0.98. As results, the current model indicates the obvious improved ability except in range 2000–3000 ppm.

**Table 3:** Evaluation value list.

ppm	Previous	3 RBs	4 RBs	5 RBs
MAE				
0-1000	269.17	146.17	144.73	147.43
1000–2000	241.19	105.88	105.05	107.32
2000–3000	384.38	63.53	60.62	50.34
3000–4000	296.37	130.36	112.23	132.58
4000–5000	219.37	132.44	129.61	130.84
5000–6000	281.91	127.54	118.67	111.01
6000–7000	217.56	139.27	123.15	123.50
7000–8000	199.44	97.01	118.71	120.33
Average	263.67	117.77	114.09	115.42
MAPE (%)				
0-1000	61.31	30.05	29.10	30.06
1000–2000	16.69	7.33	7.27	7.43
2000–3000	14.77	0.99	0.87	1.30
3000–4000	8.55	3.76	3.24	3.82
4000–5000	4.90	2.96	2.89	2.92
5000–6000	5.18	2.34	2.17	2.04
6000–7000	3.33	2.12	1.89	1.89
7000–8000	2.68	0.61	1.56	1.62
Average	14.67	6.27	6.12	6.38
RMSE				
0-1000	310.44	164.65	164.27	165.90
1000–2000	272.87	126.24	126.13	129.42
2000–3000	389.56	72.00	67.15	63.99
3000–4000	319.80	146.85	132.02	151.54
4000–5000	253.90	148.92	145.55	148.27
5000–6000	312.92	150.14	140.61	133.64
6000–7000	254.78	166.82	136.14	138.36
7000–8000	242.59	118.64	142.27	142.76
Average	294.61	136.78	131.77	134.24
CC				
0-1000	0.86	0.97	0.97	0.97
1000–2000	0.90	0.97	0.97	0.97
2000–3000	0.98	0.97	0.97	0.98
3000–4000	0.92	0.97	0.97	0.97
4000–5000	0.89	0.97	0.97	0.97
5000–6000	0.88	0.96	0.97	0.97
6000–7000	0.88	0.94	0.98	0.98
7000–8000	0.90	0.96	0.96	0.96
Average	0.90	0.96	0.97	0.97

### Advantages of our model

**Fig. 5:**
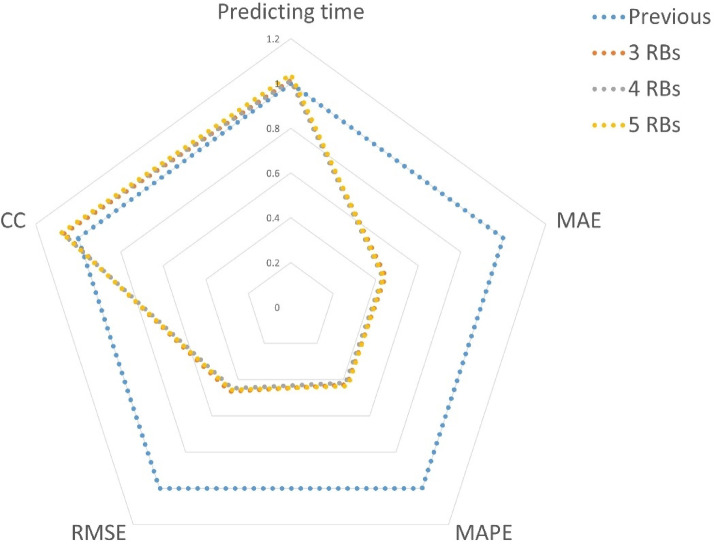
Radar chart from five evaluation metrics.

In this paper, we incorporate several critical components to improve the performance of CNN so that ours can deal appropriately with smaller measurement intervals and possess better accuracy. According to our results, we draw a radar chart from five main evaluation metrics, which is presented in Fig. 5. Notice that all results of the previous model are set to one as the base for the valuation.

From the viewpoint of predicting time, they are similar for the prediction of each sub-image and can be neglected. More specifically, although the design of RBs increases the complexity of the model, we further use the GAP layer to balance the computational cost. Next, for the other four evaluation metrics regarding accuracy, our model is obviously better, especially MAE, MAPE, and RMSE. This is the superiority of adopting the RBs and GAP layer, where the former can alleviate the vanishing gradient problem, improve feature propagation, and reduce overfitting, and the latter can facilitate parameter reduction as well as class activation mapping.

### Analysis of architectural contributions

The superior performance of the proposed CNN-SCP model is primarily attributed to the strategic integration of Residual Blocks (RB) and a Global Average Pooling (GAP) layer. Although a traditional component-wise ablation study was not performed, the experimental design and results presented in this paper already provide substantial evidence for the value of these components from two perspectives.

First, the contribution of Residual Blocks (RB) is clearly demonstrated through our sensitivity analysis on model depth. We compared model variants incorporating 3, 4, and 5 RBs. The results, as shown in Fig. 3; Table 3, indicate a consistent trend of improvement across key metrics—including prediction accuracy, MAE, RMSE, and CC—as the number of RBs increases. For instance, the 5 RBs model achieved a lower average MAE (115.42) and RMSE (134.24) compared to the 3 RBs model (MAE 117.77, RMSE 136.78). This trend clearly quantifies the value of the RB architecture in enhancing feature propagation and mitigating the vanishing gradient problem, proving that increasing network depth and complexity were critical for improving model performance.

Second, the system-level contribution of the combined RB and GAP architecture is validated by the comparison against the baseline model. The baseline model from^41^ utilizes a conventional CNN architecture without RBs or GAP. In contrast, our full CNN-SCP model achieves a quantum leap in performance (e.g., the average MAE dropped from 263.67 to 115.42). This significant gap serves as strong evidence for the effectiveness of our overall architectural design, the synergistic combination of RBs and GAP. In this design, RBs are responsible for building a deeper and more expressive feature extractor, while GAP plays a critical regularization role. By replacing traditional fully connected layers, GAP drastically reduces the number of model parameters, which effectively mitigates the risk of overfitting that might otherwise arise from the increased depth introduced by RBs, thereby enhancing the model’s generalization capability.

Overall, our findings have indirectly but strongly demonstrated the independent contribution of RBs through a sensitivity analysis on model depth and the synergistic value of the RB and GAP combination through a system-level comparison against the baseline.

### Application field

Technically speaking, reservoirs usually operate the sluice gates to release sediment and maintain storage capacity during flooding periods. The amount of high-concentration turbid water often exceeds threshold values, which renders water treatment plants incapable of processing. Therefore, by strategically deploying low-cost, high-precision instruments along the downstream river of reservoirs, it becomes feasible to issue early warnings before the arrival of high-concentration turbid water. Moreover, leveraging a robust monitoring technology allows for real-time water resource allocation. The current proposed real-time monitoring technology aligns with this objective.

**Fig. 6:**
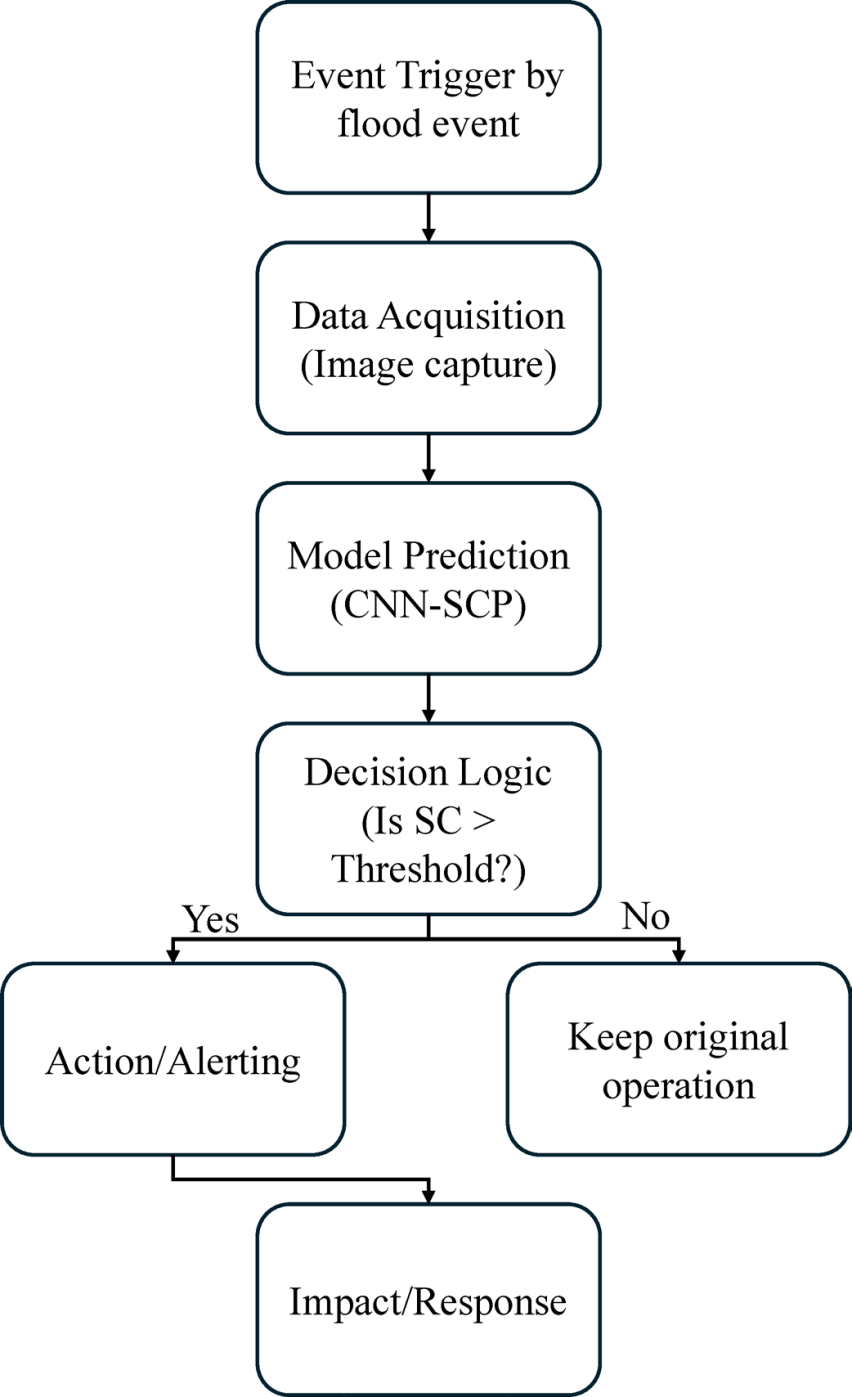
Workflow for Real-Time Sediment Monitoring and Early Warning.

Figure 6 shows a workflow for real-time sediment monitoring and early warning. Starting from a flood discharge event, it progresses through image acquisition, CNN-SCP model prediction, and a decision-making process based on sediment concentration thresholds. Depending on the outcome, the system either continues monitoring or triggers an early warning, leading to operational adjustments at water treatment facilities. This structured approach enables timely, automated responses to sediment-related risks in river systems.

## Concluding remarks

In this paper, for SC prediction, we incorporate several critical components to improve the performance of primitive CNN so that the proposed model can effectively address and deal properly with smaller measurement intervals and possess better accuracy. Specifically, introducing residual blocks helps mitigate the vanishing gradient problem and improves feature propagation by allowing identity mappings. Meanwhile, the GAP layer reduces the number of parameters, prevents overfitting, and improves generalization by replacing fully connected layers with a spatial average of feature maps, making the model more robust and computationally efficient. According to the results of the model with 5 RBs, its testing accuracy of all sub-images is almost 100%. Furthermore, the time for predicting the result of a sub-image is only 0.0490 s. This is also important for future real-time applications. The results underscore the potential of using advanced, yet accessible, CNN architectures for developing low-cost and high-precision environmental monitoring tools.

SC is a critical parameter in water resource management, particularly during turbidity surges that challenge the operational capacity of treatment facilities. Coordinated management between reservoirs and treatment plants ensures a stable domestic water supply. The proposed prediction model contributes to this need by enabling real-time estimation of SC, thereby enhancing decision support and resource allocation in water management systems.

Despite these positive results, we acknowledge several limitations. The current model was trained and validated using a dataset created under controlled laboratory conditions with consistent lighting, a uniform background, and a specific sediment type. Therefore, its performance and robustness in real-world field environments, such as complex backgrounds, diverse sediment particle sizes and compositions, and potential sensor noise, have not yet been validated. These factors could introduce visual variations not present in our current dataset, potentially affecting the model’s accuracy. Future efforts will incorporate diverse datasets from field sites to improve generalization and practical utility in dynamic, real-world conditions. Moreover, different stride values and other hyperparameters may influence the experimental results, which we also plan to consider in our future work.

## Data Availability

Data will be made available on reasonable request from the corresponding author, Che-Cheng Chang ( [checchang@fcu.edu.tw](mailto: checchang@fcu.edu.tw) ).
